# The congruence between the preferred and actual places of death among terminal cancer patients in China

**DOI:** 10.3389/fpsyg.2024.1382272

**Published:** 2024-09-10

**Authors:** Huijing Lin, Ping Ni, Bei Wu, Jing Liao, Jie Fu

**Affiliations:** ^1^School of Nursing, Tongji Medical College, Huazhong University of Science and Technology, Wuhan, Hubei, China; ^2^Rory Meyers College of Nursing and NYU Aging Incubator, New York University, New York, NY, United States; ^3^Department of Nursing, Tongji Hospital, Tongji Medical College, Huazhong University of Science and Technology, Wuhan, Hubei, China

**Keywords:** cancer, place of death, congruence, hospice care, quality of death

## Abstract

**Background:**

Dying in a preferred place is important for a good death. Currently, no study has evaluated the extent to which the preferences for the place of death (PoD) are met among terminal cancer patients in China. This study examined the congruence between the preferred and actual PoD and its predictors among terminal cancer patients in China.

**Methods:**

Between 2015 and 2023, 845 terminal cancer patients from four tertiary hospitals in Wuhan, China, were enrolled and followed till death. Face-to-face surveys at baseline and telephone-based interviews in the last month of patients’ lives were combined to learn patients’ preferred PoD. Data on patients’ actual PoD were collected from families within 1 month after patients’ death.

**Results:**

Of the 410 patients who died, 62.7% of them died in hospitals. The agreement between patients’ preferred and actual PoD was fair (*κ* = 0.221). The congruence between patients’ preferred and actual PoD was 63.0, 36.6%, and 0 for hospital death, home death, and hospice facility/nursing home death separately. Patients were more likely to die in their preferred places if their preferred place and family caregivers’ belief of patients’ preferred PoD was congruent (odds ratio [OR] = 6.464, *p* = 0.001), or if caregivers had a medically related occupation (OR = 4.577, *p* = 0.003); if patients were hospitalized at least twice in the last month of life (OR = 0.422, *p* = 0.000), or the quality of care received by patients in the last 48 h was rated good by the families (OR = 0.373, *p* = 0.011), patients were less likely to die in their preferred places.

**Conclusion:**

The congruence between patients’ preferred and actual PoD was fair. Advance care planning (ACP) needs to be popularized in China, and the quality of care in hospice facilities and nursing homes should be improved. The necessary policy support for hospice care should be made to respect cancer patients’ end-of-life (EoL) care preferences in China.

## 1 Introduction

Despite increased life expectancy and complete cures that are conceivable for many kinds of cancer, it is still the leading cause of death in 57 countries, including China (Bray et al., 2021). Terminal cancer patients occupy a large proportion of people who can benefit from hospice care focusing on easing suffering and improving quality of life at the EoL stage (Smith et al., 2012). One essential part of hospice care is to respect patients’ preferences for the place where they are cared for, spend the rest life, and die finally (Davies and Higginson, 2004). Previous literature has indicated most cancer patients all over the world preferred to die at home (Gomes et al., 2013; Teno and Curtis, 2016; Lee and Lee, 2022; Cohen et al., 2015), while the actual PoD did not necessarily match the preferred PoD. Various complications and unbearable pain may force patients and families to give up home care and seek more care from medical institutions (Kain and Eisenhauer, 2016), resulting in repeated hospitalizations and an increasing likelihood of hospital death.

Currently, more and more countries have taken steps to promote dying in preferred places through hospice care programs and ACP interventions (Van Beek et al., 2013; Driller et al., 2022), whereas such services remain relatively nascent, especially in China (Wu et al., 2016). The limited financial support, less mature management guidelines, insufficient hospice wards, and inadequate professional staff cannot satisfy the increasing needs for hospice care (Yin et al., 2017). According to the latest report on the quality of death worldwide in 2021, China ranked 53rd among 81 countries (Finkelstein et al., 2022). With a gradual increase in the incidence and mortality of cancer (Bray et al., 2021), it is necessary to promote the quality of death of terminal cancer patients in China. The congruence between the preferred and actual PoD indeed serves as a significant indicator of the quality of death (Peláez-Cantero et al., 2023).

Exploring the congruence between patients’ preferred and actual PoD as well as the associated factors can address the facilitators and barriers to the compliance of patients’ PoD preferences. Then relevant interventions can be made to follow patients’ EoL care preferences, thus promoting patients’ quality of EoL care. Nowadays, most studies related to this topic focused on the preferences for PoD (Vidal et al., 2022; Alsirafy et al., 2019; Malhotra et al., 2021; Yeung, 2020; Chen et al., 2014; Kuo et al., 2017; Gu et al., 2015; Leng et al., 2022), the actual PoD (Lee and Lee, 2022; Puechl et al., 2019; Lin et al., 2017; Tang, 2002; Lee et al., 2014; Li et al., 2020; Ding et al., 2023), or patient-family congruence on the preferred PoD (Tang et al., 2010; Tang et al., 2005; Tang et al., 2008) separately. Only very few studies explored the congruence between patients’ preferred and actual PoD among cancer patients in Brazil (*n* = 190) (Valentino et al., 2023) and Sweden (*n* = 242) (Nilsson et al., 2023). While in China, Tang et al.’s study found a poor to slight agreement between the preferred and actual PoD among 127 advanced cancer patients in Taiwan, but this study was conducted 20 years ago (Tang and McCorkle, 2003), and the policy development related to palliative care was very different between Taiwan China and China. Given the paucity of information and limited published data on the congruence between patients’ preferred and actual PoD among advanced cancer patients in China, this study sought to investigate the preferences for PoD in a cohort of relatively large-sample of different terminal cancer patients in China, to follow the patients until their death to measure the congruence between their preferred and actual PoD. Factors associated with the congruence between the preferred and actual PoD as well as the agreement in preferred PoD between patient-family caregiver dyads were also explored.

## 2 Materials and methods

### 2.1 Study design and sample

A prospective cohort study was adopted between January 2015 and August 2023 to collect data from terminal cancer patients in Wuhan, China, through face-to-face interviews and telephone-based surveys with questionnaires.

Terminal cancer patient-family caregiver dyads were recruited from four tertiary hospitals in Wuhan, China, via a convenience sampling method. Our research team members contacted the nursing administrators of four hospitals and explained the purpose and procedures of the study. The eligibility criteria for the participants included patients diagnosed with terminal cancer, unresponsive to curative therapy, competent in cognition, whose estimated life expectancy was less than 6 months, and during hospitalization. Family members identified as the primary caregivers to be involved in patients’ EoL care, able to communicate coherently, aged 18 and older, were also recruited. The exclusion criteria specified patients or caregivers who were not Chinese.

### 2.2 Data collection

Given that most Chinese work from Monday to Friday and rest on weekends in a week, it will be less busy on weekends, so the data were collected at 9–12 a.m. and 2–5 p.m. on weekends from 1 January 2015 to 31 August 2023 to allow more flexibility and respondence. After the eligibility screening in the oncology department, terminal cancer patients and family caregivers who signed informed consent or gave oral consent were face-to-face interviewed separately in an intimate room by a trained research nurse first to collect their socio-demographic information and patients’ preferred PoD. The follow-up telephone interview took place in the last month of the patients’ lives, estimated by the physician in charge to learn the patients’ preference for PoD again. At last, within 1 month after the patients’ death, families were interviewed about patients’ reception of hospice care and actual PoD by phone.

### 2.3 Ethical considerations

Ethical approval of the research protocol was granted by the institutional review board of Huazhong University of Science and Technology [registration number: [2019]IEC(S198)] as well as all the institutional review boards of the participating hospitals. All participants were informed of their voluntary participation in the study and their right to refuse to participate and withdraw from the study at any time, and all information was kept confidential.

### 2.4 Variables

#### 2.4.1 Participant characteristics

Patient-caregiver dyads’ general characteristics, such as age, gender, and the relationship between patients and caregivers (spouse, son/daughter, parents, sibling, and others), were collected at baseline. Patients’ disease conditions, such as types of cancer and the date diagnosed as cancer were also collected.

#### 2.4.2 Patients’ preferred PoD

Patients’ preferred PoD was investigated twice during a baseline face-to-face survey and follow-up telephone interview by asking them “Where is your preferred place to spend your last days of life?.” Response categories were “home,” “hospital,” “hospice facility,” “nursing home,” “relative’s home” and “other places.” Besides, family caregivers’ belief of the patients’ preferred PoD was asked by the item, “Where do you think the patient would like to spend his/her last days of life?,” with the same response categories as that of the patients’ question.

#### 2.4.3 Patients’ actual PoD

Patients’ actual PoD was obtained from family caregivers within 1 month after patients’ death. Additionally, patients’ conditions approaching death, such as whether had pain and dyspnea and whether received hospice care, were also collected from families. We also asked families to rate the quality of care that the patients received within 48 h before death and the patients’ quality of death.

#### 2.4.4 Congruence between patients’ preferred and actual PoD

The congruence was rated as “congruent” if the patients’ last reported preferred PoD was the same as their actual PoD (answered by the family caregivers). Otherwise, it was rated as “not congruent.”

### 2.5 Analysis

Data analysis was calculated with SPSS 24.0 (SPSS Inc., Chicago, IL) (IBM Corp, 2012). Descriptive statistics were used for participants’ characteristics. The *κ* statistic was used to assess the agreement between patients’ preferred and actual PoD, with values ≤0 as indicating no agreement, 0.01–0.20 as none to slight, 0.21–0.40 as fair, 0.41–0.60 as moderate, 0.61–0.80 as substantial, and 0.81–1.00 as almost perfect agreement (McHugh, 2012). Binary logistic regression models were applied to identify how significant variables were associated with the congruence. Differences at a *p*-value of <0.05 were demonstrated as statistically significant. An OR with a 95% confidence interval (CI) was calculated for each outcome variable.

## 3 Results

**Figure 1:**
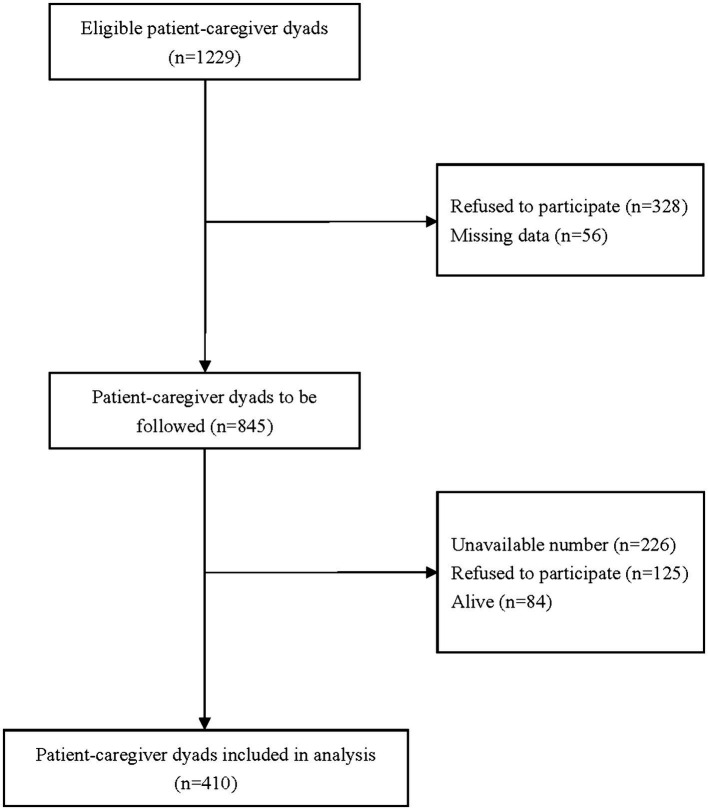
Flowchart of participant recruitment.

A total of 1,229 patient-caregiver dyads met the inclusion criteria, but 328 dyads refused to participate in the study, and 56 dyads did not complete the questionnaire, leaving 845 dyads to complete baseline tests, with a response rate of 68.8%. During the follow-up period, 84 patients were still alive until the end of the study, 125 dyads refused to continue to participate in the survey, and 226 dyads were lost to follow-up due to the unavailable phone number, leaving 410 patients who died during the study period (Figure 1).

### 3.1 Baseline characteristics of patient-caregiver dyads

The baseline characteristics of 845 dyads are presented in Table 1. Approximately half (52.3%) of the patients were male, with a median age of 54.1 years old. The most common type of cancer was lung cancer (27.5%), followed by breast cancer (17.7%), head and neck cancer (14.6%), intestinal cancer (13.0%), and gastric carcinoma (8.2%); and cancer had lasted for approximately an average of 422 days from diagnosis to study enrollment. Family caregivers were mostly the patients’ spouses (59.2%), followed by sons or daughters (27.9%). Approximately half of them were female (55.0%) with an average age of 47.2 years old.

**Table 1:** Baseline characteristics of patient-caregiver dyads (*n* = 845 dyads).

Variables	Category	*n* (%)
Terminal cancer patients		
Gender	Male	442 (52.3)
	Female	403 (47.7)
Marital status	Married/cohabited	770 (91.1)
	Unmarried/divorced/separated/widowed	75 (8.9)
Educational level	Secondary school education and below	668 (79.1)
	Bachelor’s degree or above	177 (20.9)
Hometown	Rural areas	428 (50.7)
	Urban areas	417 (49.3)
Religious belief	None	758 (89.7)
	Buddhism	41 (4.9)
	Christianity	28 (3.3)
	Others	18 (2.1)
Occupation	Non-medical-related occupation	838 (99.2)
	Medical-related occupation	7 (0.8)
Health insurance	Insured	766 (90.7)
	Uninsured	79 (9.3)
Financial status	Not adequate	485 (57.4)
	Somewhat adequate	244 (28.9)
	Adequate	116 (13.7)
Type of cancer	Lung cancer	232 (27.5)
	Breast cancer	150 (17.7)
	Head and neck cancer	123 (14.6)
	Intestinal cancer	110 (13.0)
	Gastric carcinoma	69 (8.2)
	Liver cancer	57 (6.7)
	Cervical cancer	45 (5.3)
	Ovarian cancer	17 (2.0)
	Others	42 (5.0)
Family caregivers		
Gender	Male	380 (45.0)
	Female	465 (55.0)
Marital status	Married/cohabited	763 (90.3)
	Unmarried/divorced/separated/widowed	82 (9.7)
Educational level	Secondary school education and below	604 (71.5)
	Bachelor’s degree or above	241 (28.5)
Hometown	Rural areas	417 (49.3)
	Urban areas	428 (50.7)
Religious belief	None	764 (90.4)
	Buddhism	31 (3.7)
	Christianity	22 (2.6)
	Others	28 (3.3)
Occupation	Non-medical-related occupation	819 (96.9)
	Medical-related occupation	26 (3.1)
Financial status	Not adequate	430 (50.9)
	Somewhat adequate	297 (35.1)
	Adequate	118 (14.0)
Relationship with the patient	Spouse	500 (59.2)
	Son/daughter	236 (27.9)
	Parents	45 (5.3)
	Sibling	34 (4.0)
	Others	30 (3.6)

### 3.2 EoL stage characteristics of dying patients

Most dying patients had pain (69.0%) and did not receive hospice care (89.1%). A total of 27.6% of the family caregivers rated the quality of care received by patients in the last 48 h as good, while 55.4% of them thought patients’ quality of death was good (Table 2).

**Table 2:** End-of-life stage characteristics of dying patients (*n* = 410).

Variables	Category	*n* (%)
Whether had pain in the last month	Yes	283 (69.0)
	No	127 (31.0)
Whether had dyspnea in the last month	Yes	110 (26.8)
	No	300 (73.2)
Whether received hospice care	Yes	45 (10.9)
	No	365 (89.1)
Duration of hospice care received^a^	Less than 3 days	3 (6.7)
	3–7 days	8 (17.8)
	8–30 days	14 (31.1)
	Over 30 days	20 (44.4)
Numbers of hospitalizations in the last month of patients’ life	At least twice	123 (30.0)
	Less than twice	287 (70.0)
Quality of care received by the patients in the last 48 h rated by family caregivers	Good	113 (27.6)
	Bad or not received care	297 (72.4)
Quality of death rated by family caregivers	Good	227 (55.4)
	Bad	183 (44.6)

^a^*n* = 45 for those patients who received hospice care.

### 3.3 Patients’ preferred and actual PoD

For patients’ preferred PoD, the home was selected by most patients (83.7%) in the baseline interview but decreased (68.1%) in the follow-up interview in the last month of their life. The percentage of the preference for dying in hospitals and hospice facilities increased from 9.3 to 24.4% and from 0.7 to 6.1%, respectively, in the two interviews. Additionally, the agreement in patients’ preferred PoD in patient-caregiver dyads was both fair in the baseline interview (*κ* = 0.211, *p* = 0.000) and in the follow-up interview (*κ* = 0.267, *p* = 0.000) (Table 3).

**Table 3:** Patients’ preferred place of death and congruence between patient-family caregiver dyads (*n* = 410).

(A) In the first interview										
		Family caregivers’ belief of patients’ preferred place of death							κ	*P*
		Home	Hospital	Hospice facility	Nursing home	Relative’s home	Other places	Total (%)		
Patients’ preferred place of death	Home	**274** ^a^	0.211	7	0	1	12	343 (83.7)	0.211 *^b^*	0.000^*^
	Hospital	18	**16**	3	0	0	1	38 (9.3)		
	Hospice facility	2	1	**0**	0	0	0	3 (0.7)		
	Nursing home	6	0	0	**1**	0	0	7 (1.7)		
	Relative’s home	3	1	0	0	**0**	1	5 (1.2)		
	Other places	6	3	5	0	0	**0**	14 (3.4)		
	Total (%)	309 (75.4)	70 (17.1)	15 (3.7)	1 (0.2)	1 (0.2)	14 (3.4)	410		

(B) In the follow-up interview in the last month of patients’ life										
		Family caregivers’ belief of patients’ preferred place of death							κ	*P*
		Home	Hospital	Hospice facility	Nursing home	Relative’s home	Other places	Total (%)		
Patients’ preferred place of death	Home	**236**	0.267	15	0	5	0	279 (68.1)	0.267	0.000 ^*^
	Hospital	39	**52**	7	0	2	0	100 (24.4)		
	Hospice facility	4	10	**11**	0	0	0	25 (6.1)		
	Nursing home	4	0	0	**1**	0	0	5 (1.2)		
	Relative’s home	1	0	0	0	**0**	0	1 (0.2)		
	Other places	0	0	0	0	0	**0**	0 (0)		
	Total (%)	284 (69.3)	85 (20.7)	33 (8.1)	1 (0.2)	7 (1.7)	0 (0)	410		

^a^Numbers in bold are the numbers that patients’ preferred place of death was congruent with family caregivers’ belief of patients’ preferred place of death. ^b^Kappa result is interpreted as follows: values ≤ 0 as indicating no agreement, 0.01–0.20 as none to slight, 0.21–0.40 as fair, 0.41–0.60 as moderate, 0.61–0.80 as substantial, and 0.81–1.00 as almost perfect agreement. ^*^p < 0.001.

**Figure 2:**
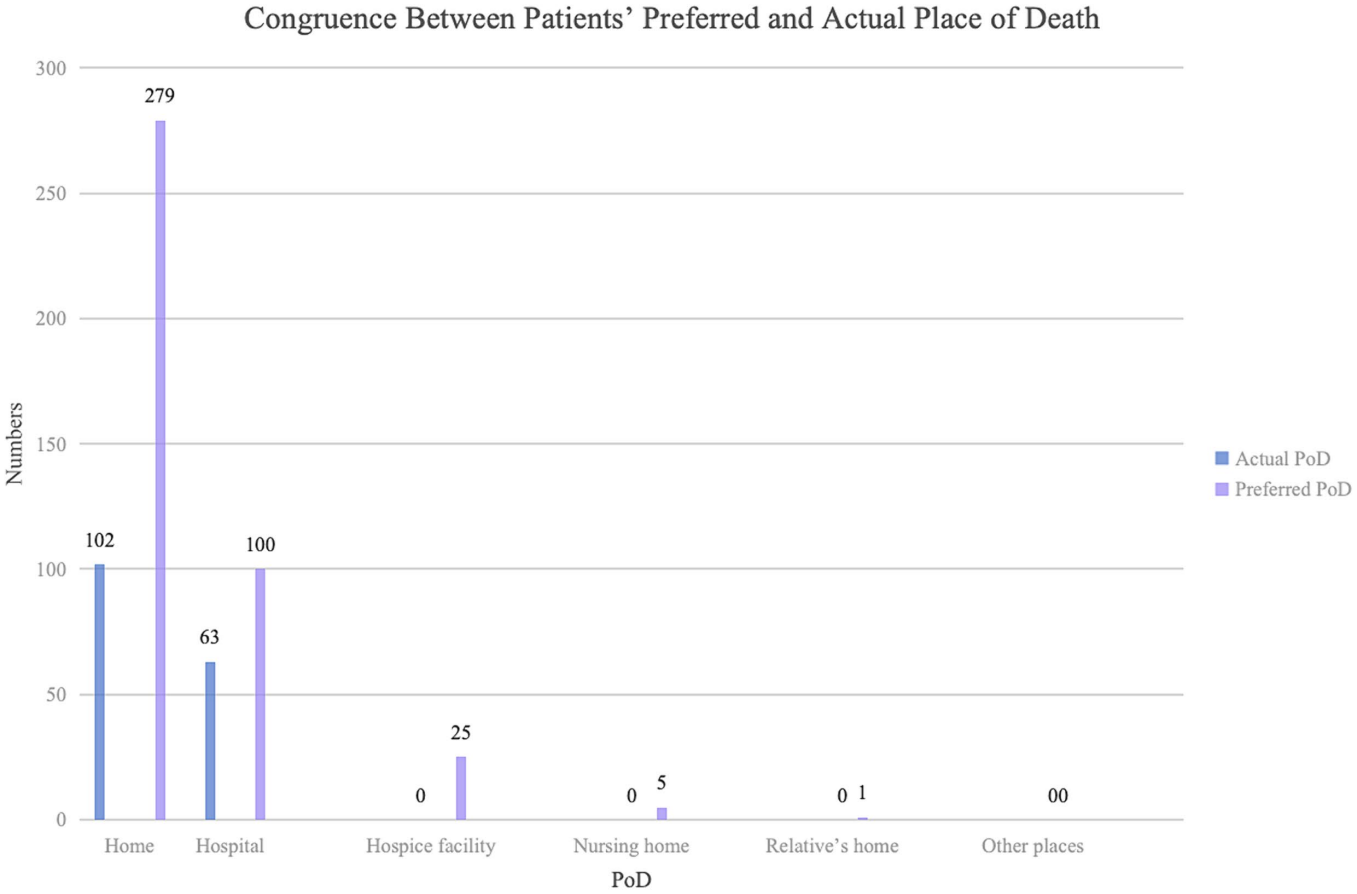
Congruence between patients’ preferred and actual place of death. PoD, place of death.

For patients’ actual PoD, most patients died in hospitals (62.7%), followed by home (36.1%) and on the way to hospitals (1.2%). The congruence between patients’ preferred and actual PoD was 63.0, 36.6%, and 0 for hospital death, home death, and hospice facilities/nursing homes/relative’s home death separately (Figure 2). In summary, the agreement between patients’ preferred and actual PoD was fair (*κ* = 0.221, *p* = 0.000) (Table 4).

**Table 4:** Congruence between patients’ preferred and actual place of death (*n* = 410).

		Actual place of death (%)							*κ*	*p*
		Home	Hospital	Hospice facility	Nursing home	Relative’s home	Other places	Total		
Patients’ preferred place of death when last asked	Home	**102 (36.6)** ^a^	172	0	0	0	5 *^b^*	279 (68.1)	0.221 *^c^*	0.000 ^*^
	Hospital	37	**63 (63.0)**	0	0	0	0	100 (24.4)		
	Hospice facility	7	18	**0 (0)**	0	0	0	25 (6.1)		
	Nursing home	1	4	0	**0 (0)**	0	0	5 (1.2)		
	Relative’s home	1	0	0	0	**0 (0)**	0	1 (0.2)		
	Other places	0	0	0	0	0	**0 (0)**	0 (0)		
	Total	148 (36.1)	257 (62.7)	0 (0)	0 (0)	0 (0)	5 *^b^* (1.2)	410		

^a^Numbers in bold are the numbers of patients who died in this specified preferred place of death. $\text{The congruence rate for}\;a\;\text{specific place of death}=\frac{\text{The numbers of patients died in their preferred place}}{\text{The numbers of patients}\;\text{who}\;\text{preferred to}\;\text{die}\;\text{in this place}.}$. ^b^*n* = 5 for those who died on the way to the hospital. ^c^Kappa result is interpreted as follows: values ≤0 as indicating no agreement, 0.01–0.20 as none to slight, 0.21–0.40 as fair, 0.41–0.60 as moderate, 0.61–0.80 as substantial, and 0.81–1.00 as almost perfect agreement. ^*^p < 0.001.

### 3.4 Predictors of the congruence between patients’ preferred and actual PoD

The congruence between patients’ preferred and actual PoD was more likely to be achieved when patients’ preferred PoD and family caregivers’ belief of patients’ preferred PoD was congruent (OR = 6.464, *p* = 0.001), or caregivers had a medical-related occupation (OR = 4.577, *p* = 0.003); if hospitalizations occurred at least twice in the last month of patients’ lives (OR = 0.422, *p* = 0.000), or the quality of care received by the patients in the last 48 h was good rated by the family caregivers (OR = 0.373, *p* = 0.011), patients were less likely to die in their preferred places (Table 5).

**Table 5:** Predictors of the congruence between patients’ preferred and actual place of death (*n* = 410).

Independent predictors (Not congruent vs. congruent)	OR	95% CI	*p*	(SE)
Congruence between patients’ preferred place of death and family caregivers’ belief of the patients’ preferred dying place				
Not congruent (reference)	1			
Congruent	6.464	2.071–20.177	0.001 ^*^	1.866
Occupation of family caregivers				
Non-medical-related occupation (reference)	1			
Medical-related occupation	4.577	1.671–12.537	0.003 ^*^	1.521
Numbers of patients’ hospitalizations in the last month of life				
Less than twice (reference)	1			
At least twice	0.422	0.268–0.665	0.000 *^†^*	−0.862
Quality of care received by the patients in the last 48 h rated by family caregivers				
Bad or not received care (reference)	1			
Good	0.373	0.175–0.798	0.011 ^*^	−0.985

CI, confidence interval; OR, odds ratio; SE, standard error. ^*^p < 0.05. ^†^*p* < 0.001.

## 4 Discussion

### 4.1 Changes in patients’ preferred PoD over time

Consistent with previous findings (Agar et al., 2008; Hinton, 1994), our study found that terminal cancer patients’ preferences for home death decreased, while the preference for dying in hospitals or hospice facilities increased as death approached. When dying is approaching, preparation for symptom control related to dying is required (National Cancer Center and National Hospice Center, 2020), while families are less confident in offering quality home care (Gao et al., 2013). In addition, this study also collected data during the COVID-19 pandemic. Considering the limited professional support outside hospitals during the COVID-19 wave, no wonder many people wanted to go to hospitals for symptom control (Lucijanic et al., 2023). Hospice care aims at relieving patients’ bad sufferings and helping them acquire a relatively comfortable dying process with dignity (Kumar et al., 2017), but such services are mostly provided in hospitals in China as professional hospice facilities are in great shortage in most areas of China, including Wuhan (Lu et al., 2018). Patients and families believe hospitals are more reliable to deal with discomfort symptoms than other settings (Li et al., 2020). Suggesting hospice services need to be expanded, not only in hospitals and hospice facilities but also provide hospice at home or nursing homes under potential legislation for dying patients to make flexible choices of preferred PoD.

### 4.2 The congruence between patients’ preferred and actual PoD

The agreement between patients’ preferred and actual PoD was fair. A total of 63.0% of those patients preferred hospital death and 36.6% of those preferred home death ultimately died in their preferred places, but the percentage was 0 for those who preferred dying in hospice facilities/nursing homes. Impacted by the Chinese principle of filial piety, compared with those patients who preferred to die in other places, patients’ wishes for hospital death were more likely to be met, as their families were more likely to make all efforts to prolong the patients’ life (Ling et al., 2020). For the home death preference, patients and families who acknowledged the Chinese proverb “falling leaves return to their roots” were more likely to return home when death was approaching (Li et al., 2020). The preference for hospice death or nursing home death was difficult to follow due to the unawareness and inaccessibility to hospice services (Wu et al., 2016; Yin et al., 2017). Additionally, hospice care fees are only partially covered by medical insurance in China (China Health Insurance, 2020); families need to pay for hospice care out of their pocket, while 57.4% of patients’ family financial status was not adequate in our study, which may be another reason why the percentage was 0 for those who preferred dying in hospice facilities/nursing homes. Additionally, the COVID-19 pandemic might also influence the changes in PoD. Shibata et al. found home death appeared in a strong uptrend while hospital death exhibited downward trends after 2019 (Shibata et al., 2024), as many were without any option stuck at home despite their wishes to be in hospital or hospice, leading to a decreased congruence between preferred and actual PoD. The fair agreement between the preferred and actual PoD suggests a need to establish and utilize community-or home-based hospice services and increase the reimbursement of hospice care under potentially considering legislation to support advance directives.

### 4.3 Predictors of the congruence between patients’ preferred and actual PoD

If patients’ preferred PoD and family caregivers’ belief of the patient’s preferred PoD was the same, patients were more likely to die in their preferred places. Previous literature has indicated that the inconsistent gap between patients and their surrogates regarding EoL care preferences can be decreased through ACP (Ke et al., 2021); in addition, ACP indeed helps patients receive care concordant with their preferences (Detering et al., 2010). Given the fact that ACP remains unfamiliar to the public and has not been regulated by legislation in China (Zhang et al., 2021), it is urgent to establish relevant policies or guidelines to promote the awareness and development of ACP in China, to increase the congruence between patients and caregivers about patients’ PoD preferences, and thus to promote the compliance of patients’ EoL care preferences.

If the family caregivers engaged in the medical-related work, patients were more likely to die in their preferred places. Family-centered decision-making process is admired in Asian culture (Haley et al., 2002). Family caregivers with medically related occupations may tend to occupy the leading position in the decision-making process and are more willing to initiate death topics with patients (Itzhaki et al., 2016), which may make them know more about the patient’s preferred PoD. Furthermore, they might be more aware that satisfying patients’ preferences was an essential indicator of the quality of good death (Peláez-Cantero et al., 2023); thus, they were more prone to respect and follow patients’ preferences. This highlights that detailed ACP or EoL care discussions should be started earlier to promote the families’ awareness of patients’ EoL preferences.

When patients were hospitalized at least twice in the last month of life, they were less likely to die in their preferred places. The more hospitalizations suggest patients may suffer more emergent symptoms that need to be addressed. Under the healthcare background in China, hospitals, especially tertiary hospitals, are the priority for patients and families to seek professional help, as most community hospitals or nursing homes can only offer basic medical care to various patients (Gu et al., 2015). Other reasons may include the less utilization of hospice facilities (Lu et al., 2018), insufficient medical resources, and weaker professional competence of nursing home staff (Gu et al., 2015), leading the patients to go to hospitals for treatment. It is important to reallocate and balance the medical services among hospitals, hospice facilities, and nursing homes, to make patients receive timely treatment in their preferred PoD.

If family caregivers considered patients had received high-quality care in the last 48 h, patients were less likely to die in their preferred places compared to those who acquired low-quality care or did not receive care. Caregivers may wish the high-quality care to last till the last moment of patients’ lives, resulting in increasing numbers of hospital deaths, whatever the patients’ preferred PoD was. For the patients who received high-quality care in hospitals in the last 48 h, they might change their preference from other places to hospital death, but this actual “last” preference was unknown to us as almost all the patients were unconscious or could not express normally at this EoL stage, suggesting to note and update patients’ EoL care preferences more dynamically. It is urgent to set up more hospice facilities in China to provide high-quality care not only in hospitals but also to improve the quality of care in hospice facilities, nursing homes, and at home, thus meeting patients’ EoL care preferences with flexibility.

### 4.4 Strengths and limitations

Our study provides insight into the congruence of patients’ preferred and actual PoD and the factors of it, as well as the agreement in patients’ preferred PoD in patient-caregiver dyads among terminal cancer patients in China for the first time with a relatively large sample size, which will help to improve compliance of patients’ EoL care preferences. There are several limitations. First, a convenience sampling method was used to recruit participants from four tertiary hospitals in Wuhan, China, possibly limiting the findings to be generalized to patients in primary or secondary hospitals, and in communities or medical care facilities. Second, patients’ preferred PoD interviewed in their last month of life may not be totally the same as the preference in the last few hours. Future research with a mixed method in different facilities could be carried out to provide more information on the congruence of patients’ preferred and actual PoD.

## 5 Conclusion

This study provides insight into the congruence between preferred and actual PoD among terminal cancer patients living in China and further highlights that if patients’ preferred PoD and family caregivers’ belief of patients’ preferred PoD was congruent, or family caregivers occupied a medical-related job, patients would tend to die in their preferred places. There are opportunities to promote the compliance of patients’ EoL care preferences, but this will require popularizing ACP and improving the quality of care not only in hospitals but also in hospice facilities and nursing homes. Policies or laws about hospice care should be made to respect patients’ rights in China, thus improving the quality of EoL care for terminal cancer patients.

## Data availability statement

The raw data supporting the conclusions of this article will be made available by the authors, without undue reservation.

## Ethics statement

The studies involving humans were approved by the institutional review board of Huazhong University of Science and Technology. The studies were conducted in accordance with the local legislation and institutional requirements. The participants provided their written informed consent to participate in this study.

## Author contributions

HL: Formal analysis, Investigation, Writing – original draft, Writing – review & editing. PN: Conceptualization, Data curation, Funding acquisition, Methodology, Project administration, Resources, Supervision, Validation, Visualization, Writing – review & editing. BW: Writing – review & editing. JL: Investigation, Writing – original draft. JF: Investigation, Writing – original draft.
